# New Drugs, Appliances, and Things Medical

**Published:** 1891-04-11

**Authors:** 


### NEW DRUGS, APPLIANCES, AND THINGS MEDICAL.

[All preparations, appliances, novelties, etc., of which a notice is desired, should be sent for The Editor, to care of The Manager, 140, Strand, London, W.C.]

### SANITARY ROSE POWDER.

Messrs. James Woolley, Sons, and Co., Market Street, Manchester, have forwarded to us samples of the above preparation, with a statement as to its composition. Our analysis proves that statement to be correct. The powder is antiseptic, soluble in water and consequently in the perspiration, hence it does not obstruct the sweat ducts and cause irritation. The colouring matters are harmless to the skin. The perfume is refreshing. The powder is well made and exceedingly fine. It is put up in three colours, pink, cream, and white. We consider it a first-class preparation, an advance in toilet antiseptics useful to all those requiring a skin powder, particularly those with delicate skin, and it will be found nice for the nursery.

### BOVININE.

Last September we noticed in full the above fluid preparation of beef, and stated our conviction that it was a valuable form of alimentation in illness or convalescence from disease. Someone has called Bovinine "The American Liebig"; we should object to the name because Liebig's Extract is not a food, but merely a condiment or relish. Now, Bovinine, on the contrary, contains from 18 to 20 per cent. of coagulable albumens; this shows that it is a food of high nutritive value. In fact, Bovinine contains not only the extractives which are to be found in Liebig's preparation, but also the large amount of albuminous material stated above. We find that it is being largely used here since its introduction, and seems likely to fill permanently a place in the physician's armamentarium. Impressed by the results of our analysis, we have tried it in several cases where difficulty of feeding occurred. In one case of scarlet fever in a child of three weeks, where milk in any form was rejected, small doses of Bovinine tided the patient over a most anxious week. In another case of gastric ulceration, where water even caused intense pain, ten days' rectal feeding with Bovinine, well diluted to relieve thirst, gave the stomach sufficient rest to enable small quantities of food to be retained. The patient in this case stated that there was less uneasiness with the Bovinine injections than with previous ones of milk, peptonised or otherwise. We might add considerably to our enumeration of cases. We are sure that Bovinine is a good preparation, and it has the advantage of always being ready and keeping well in any climate. Again, to prepare fresh beef juice is a troublesome task, and quite incapable of being properly done by the poorer classes of patients, and unless freshly prepared each time it is wanted it is useless, as it quickly becomes tainted. The use of Bovinine obviates any of these objections, and we fancy that, bulk for bulk, it is also less expensive. It can be administered in any form that the physician likes, except in combination with strong coagulants or with fluids heated much over 110 deg. F. We shall watch the results of treatment with this food with continued interest.

